# An inventory of the *Aspergillus niger *secretome by combining *in silico *predictions with shotgun proteomics data

**DOI:** 10.1186/1471-2164-11-584

**Published:** 2010-10-19

**Authors:** Machtelt Braaksma, Elena S Martens-Uzunova, Peter J Punt, Peter J Schaap

**Affiliations:** 1 TNO Quality of Life, P.O. Box 360, 3700 AJ Zeist, the Netherlands; 2 Laboratory of Microbiology, Wageningen University, Dreijenplein 10, 6703 HB Wageningen, the Netherlands; 3 Kluyver Centre for Genomics of Industrial Fermentation, P.O. Box 5057, 2600 GA Delft, the Netherlands; 4 Laboratory of Systems and Synthetic Biology, Wageningen University, Dreijenplein 10, 6703 HB Wageningen, the Netherlands

## Abstract

**Background:**

The ecological niche occupied by a fungal species, its pathogenicity and its usefulness as a microbial cell factory to a large degree depends on its secretome. Protein secretion usually requires the presence of a N-terminal signal peptide (SP) and by scanning for this feature using available highly accurate SP-prediction tools, the fraction of potentially secreted proteins can be directly predicted. However, prediction of a SP does not guarantee that the protein is actually secreted and current *in silico *prediction methods suffer from gene-model errors introduced during genome annotation.

**Results:**

A majority rule based classifier that also evaluates signal peptide predictions from the best homologs of three neighbouring *Aspergillus *species was developed to create an improved list of potential signal peptide containing proteins encoded by the *Aspergillus niger *genome. As a complement to these *in silico *predictions, the secretome associated with growth and upon carbon source depletion was determined using a shotgun proteomics approach. Overall, some 200 proteins with a predicted signal peptide were identified to be secreted proteins. Concordant changes in the secretome state were observed as a response to changes in growth/culture conditions. Additionally, two proteins secreted via a non-classical route operating in *A. niger *were identified.

**Conclusions:**

We were able to improve the *in silico *inventory of *A. niger *secretory proteins by combining different gene-model predictions from neighbouring Aspergilli and thereby avoiding prediction conflicts associated with inaccurate gene-models. The expected accuracy of signal peptide prediction for proteins that lack homologous sequences in the proteomes of related species is 85%. An experimental validation of the predicted proteome confirmed *in silico *predictions.

## Background

Fungi are heterotrophs that utilize a plethora of bio-organic carbon sources through secretion of biomass degrading enzymes. The fungal secretome is defined as the sub-proteome of soluble secreted proteins. A large part of this secretome consists of the many extracellular hydrolytic enzymes necessary to digest potential substrates. Other extracellular proteins play crucial roles in fungus-host interactions and in fungal pathogenicity. Therefore, gene classes expressed in the fungal secretome to a large degree define the ecological niche occupied by a fungal species, its impact on human health and agriculture and its usefulness as a production organism.

In the absence of direct experimental proof fungal secretomes are usually directly predicted from the genome sequence by analysing the deduced proteome for proteins with a putative N-terminal signal peptide (SP). Experimentally identified eukaryotic signal peptides on average have a sequence length between 17 and 30 amino acids and these SP are further characterized by a central hydrophobic core region of 6-15 amino acids flanked by hydrophilic N- and C- terminal regions. These features have been used to develop highly specific SP prediction tools, which all show very high prediction accuracies of 93% or higher when applied to benchmark data sets [1-3]. However, for predicted proteins the accuracy of a SP prediction heavily relies on an accurate gene-model that provides a correct N-terminal end translation of the encoded protein. Since signal peptides do not share an apparent sequence homology [2], sequence variability between secreted homologous proteins of related species is usually significantly higher at the N-terminal end. This N-terminal heterogeneity proofs to be a serious problem for homology assisted gene-finding algorithms to create a reliable gene-model useful for accurate SP prediction. Therefore, the real problem in predicting an *in silico *proteome is not the accuracy of the present prediction tools, but are the inaccurate gene-models used as input for these tools. Furthermore, a number of proteins with a correctly predicted SP are in reality not secreted, for instance because they are resident ER proteins [4]. Thus, in the absence of direct experimental proof of secretion, an *in silico *predicted secretome does not correctly represent the actual secretome.

The genus *Aspergillus *represents an important group of filamentous fungi with significant impact on many facets of human welfare. Recently, genome-sequencing projects of at least 10 *Aspergillus *species have been completed or are nearing completion. The corresponding proteomes are usually inferred from gene-models derived with automated gene prediction tools. Consequently, the large majority of the predicted protein coding sequences are hypothetical and have a variable degree of accuracy. An encouraging exception is the extensively manually annotated genome sequence of *A. niger *[5]. Genome sequences are publicly available from two *A. niger *strains [5,6], which allows for a direct cross-validation of genome data and for a direct comparison of most of the independently derived gene-models. *A. niger *is an excellent producer of a suite of extracellular enzymes and many of them have been granted a GRAS (Generally Recognized As Safe) status by the U.S. Food and Drug Administration (FDA). These properties have made this fungus a preferred production organism for a range of secreted commercial enzymes. Among the most important of them are amylases, asparaginases, beta-galactosidases, glucose oxidase, glycosidases, lipases, phospholipases, proteases, phytases and several hemicellulases [7]. Nevertheless, based on the recently elucidated genomic sequence of *A. niger*, it can be estimated that currently direct experimental proof of secretion of only a fraction of the potentially secreted proteins exists.

In this study we combined comparative *in silico *SP predictions for classically secreted proteins with an extensive set of experimental secretome data derived from mass spectrometry analysis of *A. niger *secretome enriched fractions. Cross-species validation of *in silico *SP predictions produced a more accurate list of potentially secreted proteins and an improved annotation of the underlying gene-models. The secretomes of *A. niger *associated with growth on sorbitol and galacturonic acid and upon depletion of the carbon source were analyzed using a shotgun proteomics approach. This analysis provided insight into the dynamics of the *A. niger *secretome and direct experimental proof of secretion for known and unknown signal peptide directed proteins (SP proteins).

## Results and Discussion

### *In silico *prediction and validation of the secretome of *A. niger*

To estimate the prediction accuracy of an *in silico *secretome prediction, we compared *in silico *SP predictions from two *A. niger *strains (*A. niger *CBS 513.88 and *A. niger *ATCC 1015), and further compared them with SP predictions of orthologous proteins from three closely related functionally annotated *Aspergillus *species (*A. oryzae *RIB40 [8]*, A. fumigatus *Af293 [9], and *A. nidulans *FGSC A4 [10]).

### Cross-validation of SP predictions between *A. niger *CBS 513.88 and *A. niger *ATCC 1015

The genome of the industrial production strain *A. niger *CBS 513.88 was recently sequenced [5] and a total of 14.086 protein coding genes (CDS) were identified. Of these CDS 191 are known to be N-terminally truncated, because the corresponding loci are located at a contig border. When the signalP3 signal peptide prediction suite [2] is used, a classical signal sequence for secretion is detected in at least 1831 predicted proteins (Table 1). For reasons argued above, this *in silico *prediction is not very accurate, because it depends heavily on the correctness of the underlying gene-models.

**Table 1:** Proteome size and single signalP3 signal peptide and signal anchor predictions of four selected *Aspergillus *species

Species	*A. niger *CBS 513.88	*A. niger *ATCC 1015	*A. oryzae *RIB40	*A. fumigatus *Af293	*A. nidulans *FGSC A4
**protein CDS**	14086*	11197**	10406**	9887**	10665**
**signalP3 NN**	1831	1540	1751	1258	1469
**signalP3 HMM SP**	2016	1687	1802	1067	1612
**signalP3 HMM SA**	627	529	582	391	488

Species are ranked by their phylogenetic distance to *A. niger *CBS 513.88

NN, neural network method (proteins were considered to be SP proteins if the signalP3 *D*-score >0.43); HMM, hidden Markov model; SP, signal peptide; SA, signal anchor

* Number obtained from the Refseq section of GenBank

** Numbers obtained from http://www.broad.mit.edu/annotation/genome/aspergillus_group/MultiHome.html

The genome of *A. niger *strain ATCC 1015 was annotated independently and is predicted to encode some 11.200 protein encoding genes [6]. The signalP3-NN neural network algorithm predicts that 1540 *A. niger *ATCC 1015 gene-models encode proteins with a SP (Table 1). In total 1257 of those gene-models are orthologous to a single CBS 513.88 gene-model and are undoubtedly derived from the equivalent locus. This subset was used to compare the SP prediction results. In as much as 30% of these predicted proteins conflicting signalP3-NN prediction results were obtained due to alternative start codon selection (Additional File 1).

### Cross-validation of SP predictions using other Aspergilli as classifier species

To improve the precision of the *A. niger *whole proteome SP prediction, signalP3-NN prediction results of the *A. niger *CBS 513.88 proteome were also compared to those of the best homologous proteins of three closely related fully annotated *Aspergillus *species, i.e. *A. oryzae *strain RIB40 [8]*, A. fumigatus *strain Af293 [9], and *A. nidulans *strain FGSC A4 [10]. A summary of the single genome signalP3 predictions of these *Aspergillus *sp. is presented in Table 1.

At such a close phylogenetic distance, clusters of orthologous proteins not only are predicted to have the same molecular function in the different species, but also are expected to exert this molecular function at the equivalent location. If this is true, SP prediction results derived from individual signalP3 predictions for *Aspergillus *sp. proteins orthologous to an *A. niger *protein of interest can be used as an independent majority rule based classifier. The classifier was constructed in the following way. For each genome the complete list of predicted SP proteins and their reciprocal top BlastP hits with *A. niger *CBS 513.88 proteins were sorted into *A. niger *centred orthologous protein clusters as is detailed in Methods. Subsequently, each cluster member was also screened for a possible signal anchor (SA). In this way 1527 *A. niger *centred orthologous clusters with at least one putative SP protein could be formed. Of these clusters 1274 are spanning three to five genomes and 253 are formed by bidirectional "best hit" protein-pairs (Additional File 1). In total 669 thus formed protein pairs and clusters showed a pan-genomic cross-validation of SP prediction.

It should be noted that not all of these cross-validated proteins are actually secreted. Proteins with a function in the secretion pathway or related compartments such as the vacuole may with this *in silico *approach be classified as (potentially) secreted proteins. For instance, we have observed clustering of at least 12 resident ER proteins, which can be recognized by the presence of a C-terminal ER-retention motif [4] (Additional File 1). However, as for most of the here classified proteins a molecular functional characterization is lacking, we have not taken this into account in our analysis. A further inspection of Additional File 1 suggests that for all five analysed *Aspergillus *sp. the accuracy of a single genome *in silico *SP prediction is approximately 85%.

### Improved annotation of *A. niger *gene-models

In 33 protein clusters of the classifier an *A. niger *CBS 513.88 protein predicted to be a non-SP protein was clustered exclusively with classifier SP proteins being orthologous proteins from the other *Aspergillus *species. Four of those likely false negative signalP3 predictions were re-evaluated by aligning their N-terminal ends (Additional File 2). In all cases selection of an alternative start codon in the most likely reading frame would *i*) bring the predicted protein sequence length in better agreement with the predicted protein sequence length of the close by orthologs and *ii*) add a predicted signal peptide feature to the alternative N-terminal end of the predicted CBS 513.88 protein.

Vice versa, in 55 cases a SP prediction for an *A. niger *CBS 513.88 protein was not supported by predictions for the orthologous classifier proteins in 55 of the protein clusters. While the molecular function prediction of most of them clearly suggests an intracellular molecular function, in some cases the classifier also showed an ambiguous behaviour in separating SP and SA predictions. For instance, the protein sequences of An15g01200 (*A. niger *CBS 513.88) and the equivalent protein 137591 (*A. niger *ATCC 1015) differ both in length and in their SP/SA prediction. However, compared to the best homologs of the other *Aspergillus *sp. both proteins appear to be N-terminally truncated and therefore both should be N-terminally extended. A screen of *A. niger *ATCC 1015 EST sequence data available at the Broad institute (http://www.broadinstitute.org/) demonstrated the presence of an alternative start codon revealing a putative SA with a probability of 0.993 for the newly inferred protein (see Additional File 2 for details).

### Proteogenome analysis of secretome enriched fractions

**Figure 1:**
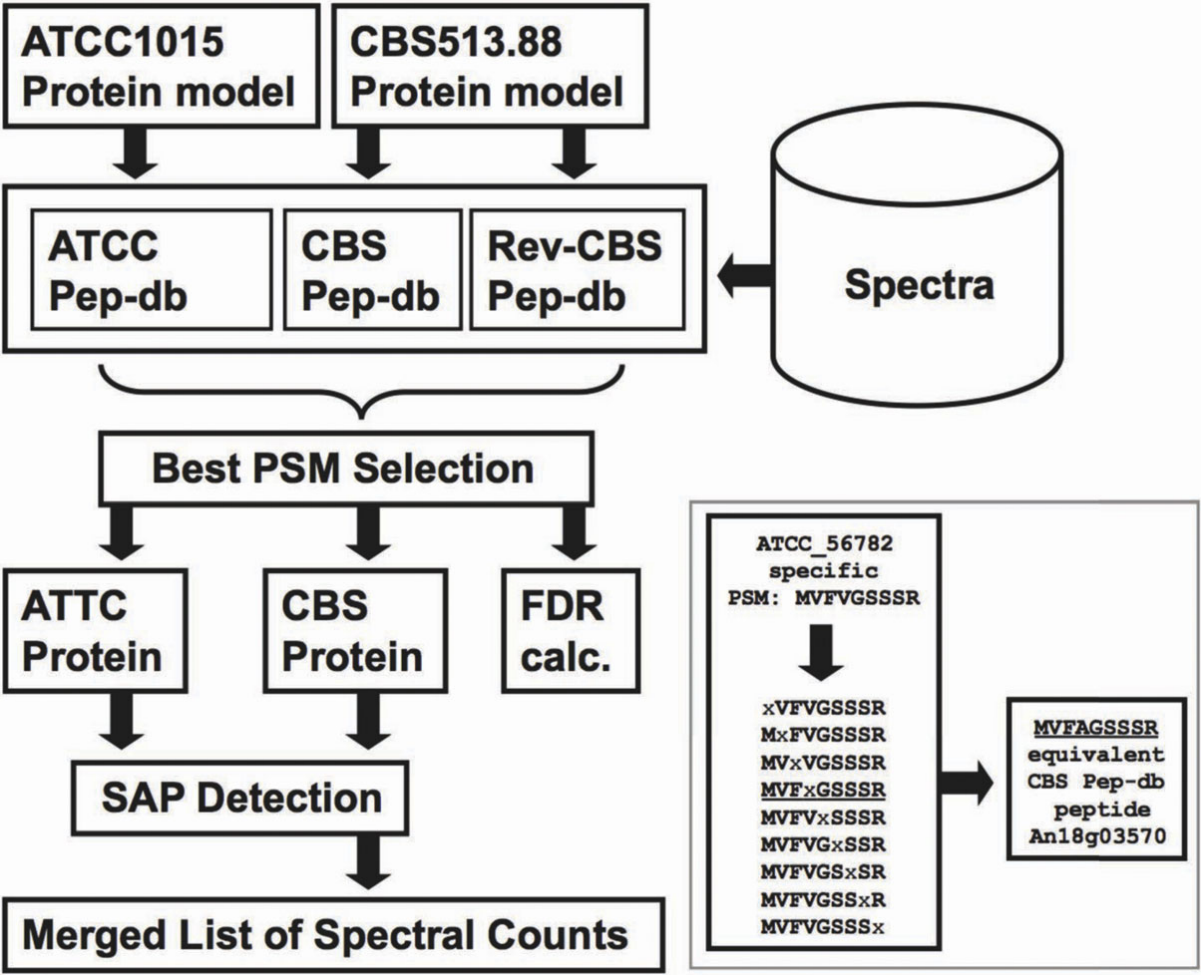
**Schematic of the Spectrum to Peptide matching pipeline**. Forward and reversed (REV-CBS) databases were searched with local implementation of the OMSSA MS/MS search engine. Threshold Expect values for matching peptides were estimated from the false discovery rate (FDR). Best Accepted peptide-spectrum matches (PSM) selection was done by ranking for each MS/MS spectrum the output of each individual peptide database by *E*-value and selection of the top hit identified peptide sequence. Insert: Detection of a single amino acid polymorphism (SAP). A wildcard character (x) is introduced at each position of a single proteome matching peptide, followed by a pattern search in the complementary proteome. In the given example using the ATCC 1015 single proteome matching peptide as a template, a single equivalent peptide, derived from An18g03570 is retrieved from the complementary proteome. An18g03570 is 99% identical to ATCC 1015 protein 56782.

Secretome enriched fractions of *A. niger *N402 cultured under controlled conditions in defined synthetic media were analyzed by high-throughput mass spectrometry (see Methods). The culture supernatants of three conditions were analyzed. In the first two conditions, samples of secretome enriched fractions grown on sorbitol were compared with samples from secretome enriched fractions grown on galacturonic acid (GalA). For induction of the pectinolytic system sorbitol is considered to be a neutral carbon source, while the carbon source GalA is the major constituent of pectin and a specific inducer [11,12]. In the third condition, prolonged carbon source exhaustion was exploited. The Open Mass Spectrometry Search Algorithm (OMSSA) search engine [13] was used for the analysis of these tandem mass spectra. One of the major causes for errors in protein identification is incompleteness of the peptide sequence database due to missed protein encoding genes and gene-models errors. Therefore, tandem mass spectra obtained by shotgun proteomics of the enriched secretome fractions were independently matched with peptide databases derived from the predicted proteome sequences of both *A. niger *strains. To quantify false positive rates of peptide identification, all spectra were also independently searched against a reverse peptide database constructed from the reverse *A. niger *CBS 513.88 proteome (see Methods). At the selected *E*-value threshold < 0.01 for acceptance of a PSM, the spectrum level FDR was limited to 2% or less under all conditions. The bioinformatics analysis workflow is presented in figure 1. The full list and functional annotation of thus identified proteins and the conditions under which they were detected are shown in Additional File 3.

The genome of *A. niger *CBS 513.88 has been subject of an extensive molecular function prediction, followed by thorough manual verification. As a result, the genome sequence of this strain encompasses a higher number of protein-coding genes compared to *A. niger *ATCC 1015. Therefore, the CBS 513.88 proteome was chosen as the primary database for further analysis. Overall, 7523 accepted PSMs identified 285 predicted *A. niger *CBS 513.88 proteins. Additionally, we detected 7 more *A. niger *ATCC 1015 proteins with no apparent matching locus in the genome of the other strain (Additional File 3). Conversely, 25 identified *A. niger *CBS 513.88 proteins lacked an *A. niger *ATCC 1015 gene-model, even though in most cases the corresponding locus was present in the ATCC 1015 genome.

Wright *et al*. [14] and Tsang *et al*. [15] used a similar shot-gun proteomics procedure to exploit the *A. niger *proteome. Very recently also an *A. niger *proteome study based on 2D-gel electrophoresis was carried out by Lu *et al. *[16]. In the study of Wright *et al. *[17], where frozen mycelium was used as study material, 214 different loci were identified. As expected by the differences of source material the overlap with the present study is limited to only eight proteins. In the study by Lu *et al. *[19] about 70 proteins were detected in the secretome of which the majority was also found in our data set. From these, only 3 SP proteins were not identified in our data set. Similarly to our results, the shot-gun proteomics approach from Tsang *et al. *[18] identified about 200 secretome-associated proteins, from which the large majority correspond to *in silico *classified SP proteins, confirming the validity of our approach. About 40 of the proteins identified by Tsang *et al. *were not identified in our data set, whereas our experimental data set identified more than 80 SP proteins not identified by Tsang *et al*. [18].

Peptide spectrum matching requires a high quality proteome. While by and large correct, gene-model predictions may suffer from exon-identification and exon-border errors, leading to a mismatch with identified peptide spectra. Another reason for not obtaining completely matching peptide spectra may be due to the presence of genetic variation, small strain differences leading to single amino acid polymorphisms between the investigated strain *A. niger *N402 and the two annotated *A. niger *genomes used for mass analysis. In a systematic analysis of matching peptides that are only present the peptide databases of the annotated genomes, 31 single proteome peptides were found to match with a single amino acid polymorphism in the equivalent protein of the other strain. The large majority of these amino acid polymorphisms (29 out of 31) was observed between strain CBS 513.88 and strain N402, suggesting that strain N402 is more closely related to ATCC 1015.

### Functional analysis of secretome enriched fractions

Fungal secretome enriched samples are expected to contain a complex mixture of possibly hundreds of SP proteins with a minimal contribution of proteins acquired through cell lysis.

A simple differential measure of relative protein abundance known as 'spectral counting' can be used to quantify the relative contribution of each protein to this mixture. It has been shown that the total number of spectra that identify peptides originating from a given protein shows good linear correlation with the abundance of that protein [17,18] and a good sensitivity for detecting changes in protein abundance [19,20]. The major analytical caveats to using this approach is that spectral count ratios can be biased by undersampling, the fact that different peptides have different physiochemical properties that affect MS detection, and that in complex mixtures for proteins with a low number of spectral counts this correlation is not very strong [19].

**Figure 2:**
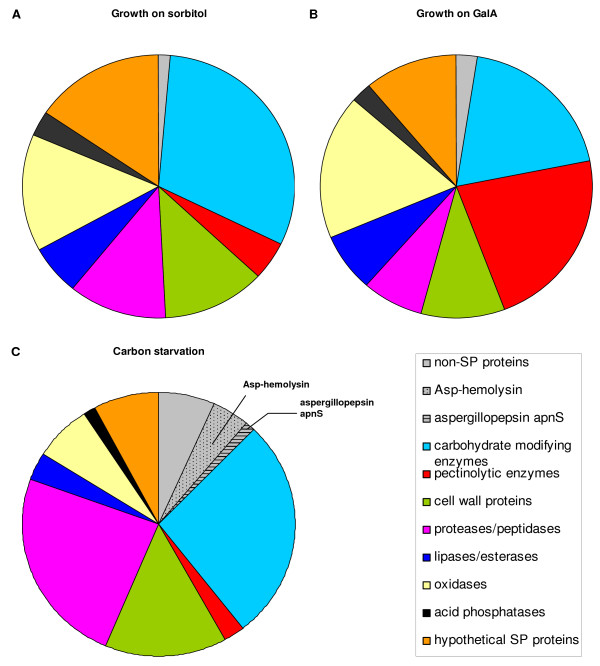
**Categorization of the *A. niger *secretome**. Detected secretome when grown in sorbitol (A), in galacturonic acid (B), or under carbon starvation conditions (C). For each condition, the contribution of a protein to a category was normalized based on the total number of spectra.

To overcome such limitations in interpreting relative presentation of proteins, functional annotation clustering was used to identify biological processes overrepresented among the proteins detected in the enriched secretome fractions. For this, detected proteins were clustered in nine groups. Seven groups were based on molecular function prediction by using the FunCat annotation scheme [21] and the predicted molecular function as guidance. Functionally unclassified proteins with an SP prediction and a functionally diverse group of "non-SP proteins" formed two additional groups (figure 2). The group "C-compound and carbohydrate metabolism" (CH) together with the enzymes of the pectinolytic system formed the largest functional annotation cluster. From figure 2 it is obvious that compared to growth on sorbitol the pectinolytic system is induced upon growth on GalA. Therefore "pectin-modifying proteins" were put in a cluster separate from the CH cluster. FunCat category "extracellular protein degradation" was used as a basis for the cluster "protein and peptide degradation". Furthermore, we distinguished "cell wall components", "oxidases", "lipase-esterases" and "acid phosphatases".

Overall, 98% of the 2722 accepted PSMs obtained from the sorbitol samples could be traced back to a SP protein in one of the seven functional annotation clusters or the hypothetical SP protein cluster. Almost identical results were obtained for the GalA samples. For the carbon source starvation conditions this amounted to 88% of the accepted PSMs (figure 2 and Additional File 3). These results suggest that the quantitative contribution of cell lysis to the secretome enriched fractions demonstrated by the detection of an array of functionally diverse non-SP proteins is indeed limited. The contribution of non-SP proteins seems to be significantly higher in secretome enriched samples derived from starvation conditions, but this difference is primarily caused by the specific expression of a single non-SP protein An01g09980, with a strong similarity to Asp-hemolysin from *A. fumigatus. *Asp-hemolysin has been purified from the culture filtrate of *A. fumigatus*, while no SP is detected [22]. The fact that the *A. niger *homologous protein is detected in significant amounts in the culture filtrate as well, suggests that this is a non-classically secreted protein. If the Asp-hemolysin is indeed intentionally secreted, the relative contribution of cell lysis in secretome enriched fractions under starvation conditions is much more comparable to what is observed for sorbitol and GalA.

More than 98% of the here-identified secreted proteins are supported by both signalP3 and majority-rule predictions. However, the list also includes an ATCC 1015 protein (128537), which is supported by the rule based classifier based prediction only. Others, such as An02g11390, are ambiguous in their signalP3 and classifiers based SP predictions, but are clearly present in secretome enriched fractions. If we consider these proteins to be genuinely secreted proteins the contribution of cell lysis in our data set is even lower than discussed above.

### Carbohydrate modifying enzymes

Three controlled fermentation conditions were chosen to study the relative contribution of various classes of carbohydrate modifying enzymes and proteases to the secretome. To minimize the effect of undersampling, sorbitol, GalA and starvation-specific samples were pooled. Between the three conditions significant changes were observed for all functional annotation clusters, except for the cluster of acid phosphatases. Upon growth on GalA "pectinolytic enzymes" are overrepresented. In contrast, proteins present in the CH cluster are overrepresented upon growth on sorbitol.

Although some pectinolytic enzymes are found in all sampled secretome fractions, pinpointing to constitutive expression [12], the pectinolytic system is strongly induced under GalA growth conditions. Compared to growth on sorbitol not only the number of spectral counts related to the pectinolytic functions increase upon growth on GalA, but also the diversity of enzymatic functions. To identify *A. niger *genes potentially involved in galacturonic acid catabolism, we have previously compared *A. niger *N402 microarray data obtained upon growth of the fungus on various carbon sources. Fifteen highly correlating genes were found that were specifically induced on galacturonic acid [11]. GalA specifically activates the transcription rate of six extracellular enzymes. In the GalA derived secretome five of those, pectin lyase A, three exoPGs (PGAX, PGXB and PGXC) [23] and An02g02540, a putative pectin acetylesterase, are detected, but only under this condition. An08g01710, a putative alpha-arabinofuranosidase with no apparent SP and part of this transcriptional cluster, is not found in any of the secretome fractions (Table 2).

**Table 2:** Pectinolytic enzymes with a correlating transcriptional profile in galacturonic acid transfer cultures in secretome enriched fractions

Locus tag	r *	Gene name	Molecular Function	Signal Peptide	Spectral counts		
					Sorbitol	GalA **	Starvation
An14g04370	0.999	*pelA*	Pectin lyase A	Yes	0	8	0
An12g07500	0.979	*pgaX*	Exopolygalacturonase X	Yes	0	18	0
An11g04040	0.978	*pgaA*	Exopolygalacturonase A	Yes		Not detected	
An03g06740	0.971	*pgxB*	Exopolygalacturonase B	Yes	0	40	0
An02g12450	0.964	*pgxC*	Exopolygalacturonase/exoxylogalacturonan hydrolase	Yes	0	19	0
An08g01710	0.953		Putative arabinofuranosidase	No		Not detected	
An02g02540	0.963		Putative pectin acetylesterase	Yes	0	19	1

* *r*, correlation coefficient (data from [11]); ** GalA, galacturonic acid

Glucan is one of the major chemical components of the *Aspergillus *cell wall and 1,3-beta-glucanosyltransferases therefore play an active role in fungal cell wall biosynthesis [24]. Overall eight 1,3-beta-glucanosyltransferase genes from the GH72 family are present in the *A. niger *genomes. All eight encoded proteins are predicted to have a Glycosylphosphatidylinositol (GPI) anchor, that becomes linked to the C-terminal residue after a proteolytic cleavage occurring at the so-called ω-site [25]. A multiple alignment of the eight encoding protein sequences suggest that they can be assigned to three subgroups (Table 3). Four of those 1,3-beta glucanosyltransferases representing each of these subgroups are detected in the three main secretomes.

**Table 3:** Expression of 1,3-beta-glucanosyltransferase genes present in the A. niger genome

Group	Ordered locus name	Signal peptide prediction		GPI-anchor Prediction**
		signalP3*	Classifier	
1	**ATCC_53033** An02g03070	Yes Yes	Yes Ambiguous	Highly probable Highly probable
2	An02g09050, **An08g07350** **An10g00400**	Yes Yes Yes	Yes Yes Yes	Probable Highly probable Highly probable
3	**An09g00670** An03g06220 An16g06120	Yes No Yes	Yes Yes N.A.	Highly probable Probable Highly probable

* SignalP3 algorithm [2]

** using the PredGPI algorithm [25]

Bold and underlined: proteins are detected by mass spectronomy in the secretome enriched fractions

N.A.: Not available, classifier predictions are not valid, because the cluster-size is too small

### Proteases

Carbon source starvation conditions were chosen to induce extracellular proteases. Indeed, where the fraction of spectral counts assigned to "extracellular protein degradation" is 7% and 12% for growth conditions GalA and sorbitol, respectively, under starvation conditions this is 24%. The extracellular aspartic proteinase aspergillopepsin I (PepA) is by far most abundant under starvation conditions. Other high abundant proteases are An08g04490 [26], and the putative serine proteases An14g02470, An06g00190, and An03g05200, which together with PepA account for over 75% of the PSMs assigned to proteases under starvation conditions (Table 4). In addition, under all tested conditions, a protease (53364) was detected specific to *A. niger *ATCC 1015 locus only. This aspartic-type endopeptidase has a predicted SP and homologs are widespread in the genomes of other *Aspergillus *sp.

**Table 4:** Proteases detected in secretome enriched fractions of *A. niger *N402 cultured under a set of controlled conditions

MEROPS family	Locus tag	Spectral counts		
		sorbitol	GalA	starvation
Peptidase family A1 (pepsin family)	An01g00370*	0	0	27
	An02g07210	0	0	2
	An04g01440	0	0	2
	An12g03300	0	1	0
	An13g02130	1	1	0
	An14g04710	87	33	226
	An15g06280	4	0	0
	An18g01320	9	8	17
	ATCC 53364	2	3	4
Peptidase family M28 (aminopeptidase Y family)	An03g01660	0	8	3
Peptidase family S10 (carboxypeptidase Y family)	An02g04690	9	5	3
	An03g05200	38	21	49
	An14g02150	0	1	4
Peptidase family S28 (lysosomal Pro-Xaa carboxypeptidase )	An08g04490	58	25	53
	An12g05960	42	20	29
Peptidase family S53 (sedolisin family)	An01g01750	14	5	24
	An03g01010	9	10	2
	An06g00190	7	16	68
	An08g04640	17	4	24
	An14g02470	18	18	67

* For An01g00370 no signal peptide could be predicted

Overall, 20 proteases were identified in this study (Table 4), of which all but An01g00370 have a SP prediction. An01g00370 is an aspartic protease with strong similarity to aspergillopepsin ApnS of *A. phoenicis*, and is only detected under starvation conditions. An01g00370 is not a protein directed by a classical signal peptide for secretion nor can such signal peptides be detected in orthologous (predicted) proteins from other *Aspergillus *sp. Nevertheless, the number of spectra derived from this protein is relatively high (Table 4), making it unlikely that this protein was detected in these fractions due to lysis. Therefore, in addition to the highly expressed putative hemolysin homolog, this protease is the second likely candidate for non-classical secretion and indeed, when subjected to the Secretome P2.0 algorithm [27], both protein sequences score above the set threshold value for non-classical secretion. Furthermore, disruption of this protease results in a significant increase of the secreted level of heterologous laccase activity [28], suggesting it is in fact a functional extracellular protease secreted by non-classical routing.

## Conclusions

In this work we present an improved list of SP proteins encoded by the *A. niger *genome. The list of SP proteins as predicted by signalP3 was improved by the additional implementation of a rule based classifier constructed from single genome signalP predictions of the best homologs combined with a simple decision rule. Conflicting SP predictions are mostly due to inaccurate gene-models. Re-evaluation of the CDS by N-terminal alignment showed that selection of an alternative start codon in the same reading frame is in most cases sufficient to obtain an agreement. For putative SP proteins that do not have clear homologs in the proteomes of the related species and thus depend on signalP predictions only, an accuracy of 85% can be expected. Proteogenome analysis of secretome enriched fractions subsequently provided experimental evidence for secretion of at least 209 of these predicted SP proteins in our data set, whereas about 40 additional predicted SP proteins were identified in the data sets from Tsang *et al*. [18] and Lu *et al *[19].

The *A. niger *secretome responds dynamically to changes of the carbon source. The majority of the detected carbohydrate modifying enzymes are present under both sorbitol and GalA growth conditions. However, the relative contribution of the individual enzymes significantly changed with the carbon source. As was already evident from transcriptome data [11,12], the pectinolytic system is most strongly induced under the GalA growth conditions, where 22 of the 30 proteins are either solely present or significantly more abundant in samples from GalA cultures. The most prominent difference between the growth and starvation conditions is the relative contribution of a number of abundant proteases which levels increase even further under starvation conditions. However, a few other proteases are exclusively detected upon growth on sorbitol or GalA.

Although a broad spectrum of non-SP proteins was identified in the secretome enriched fractions, the relative contribution of lysis was very limited, even under starvation conditions. Still, relative high concentrations of, two non-SP proteins with a putative extracellular function, An01g09980 and An01g00370, were detected. Most probably these proteins are exported outside the cell by active transport mechanisms, indicating that a non-classical secretion pathway operates in *A. niger. *Further experimental validation of this pathway will be required by more detailed analysis of trafficking of these proteins.

## Methods

### Bioinformatics

Signal peptide predictions: SP predictions were done with a local implementation of the signalP3 NN and HMM algorithms [2]. Proteins were considered to be SP proteins if the signalP-NN *D-*score was higher then 0.43. Additional signal anchor predictions were done with a local implementation of the signalP3-HMM algorithm.

Signal peptide cross-validation and construction of the classifier: the predicted proteomes of *A. niger *strains CBS 513.88 and ATCC 1015, *A. oryzae *RIB40, *A. fumigatus *AF293 and *A. nidulans *FGSC A4 were used as input. For each individual protein a SP prediction was done using the signalP3 algorithm. If the score was above the set threshold *D*-value the tag SP was added to the ordered locus name. Next, a bidirectional Blastp [29] was done between the *A. niger *CBS 513.88 proteome and the proteome of *A. niger *ATCC 1015, and between the *A. niger *CBS 513.88 proteome and the proteome of the three other *Aspergillus *sp. Each set of pair wise tabular outputs was stringently parsed for bidirectional best hit pairs using the following criteria: *i*) between the two sequences the percentage of identity must be above a set threshold level of 40%, *ii*) the two aligned protein sequences must be of similar size (a difference in size of less than 20% was accepted) and *iii*) the aligned region must include more than 70% of the smallest protein sequence. Next, these bidirectional best hits were used to form *A. niger *centered protein clusters. Protein clusters with at least one SP-tag added to an ordered locus name were selected for the construction of the classifier. Implementation: In comparison with single genome signalP3 predictions deviating classifier SP predictions were considered to be of better-quality when the two following criteria were met *i*) a cluster-size of at least three species and *ii*) between the non-*A. niger *classifier proteins a complete agreement in SP prediction. Muscle [30] was used for protein multiple sequence alignments. PredGPI [25] was used for GPI-anchor predictor of putative 1,3-beta-glucanosyltransferase genes. The general prosite consensus pattern was used to identify C-terminal ER retention motifs in predicted SP proteins.

Mass spectrometry data analysis. The 98.150 MS/MS spectra resulting from MS analysis of the *A. niger *secretome enriched samples (see below) were submitted to a local implementation of the OMSSA search engine [13]. MS/MS spectra were independently searched against peptide databases derived from the predicted proteomes of *A. niger *strain CBS 513.88 and of strain ATCC 1015 and against a database of randomized sequences constructed from the reverse of the CBS 513.88 proteome. All OMSSA searches used the following parameters: a precursor ion tolerance of 0.03 Da, fragment ion tolerance of 0.5 Da, a miss cleavage allowance of up to and including 2, all cysteines were considered to be carboxyamidomethylated, oxidation of methionine and deamination of glutamine and aspargine were treated as variable modifications.

The set *E*-value threshold was determined iteratively from the false discovery rate (FDR) and was set to 0.01. With this setting an FDR of < 2% was obtained for all samples.

FDR calculation was done as follows: for each identified spectrum with a threshold *E*-value < 0.01 accepted peptide-spectrum matches (PSM) with each individual peptide database were ranked by their *E*-value and the top hit identified peptide sequence was selected. The FDR was calculated from top hit spectral matches to peptides in the reversed database as described by Elias and Gygi [31].

The data is available in the PRIDE database [32] (http://www.ebi.ac.uk/pride) under accession numbers 13662, 13663, 13664 and 13665.

### Culture conditions

The fungal strain used in this study was *A. niger *wild type N402, *cspA1 *(conferring short conidiophores) a derivative of ATCC 9029 (alternative names NRRL 3, CBS 120.49, N400)

Conditions for growth on sorbitol and galacturonic acid: For pre-culture, 1.0 × 106 spores per millilitre were inoculated into 2.5-L fermentors (Applikon) containing 2.2 L of minimal medium [33] with 0.05% yeast extract and either 50 mM D-sorbitol or 50 mM D-galacturonic acid as carbon source, at 30°C and pH 3.5. Spore germination in bioreactors was as described previously [34], with headspace aeration and a stirring speed of 300 rpm, and when dissolved oxygen levels were below 60%, stirring speed was changed to 750 rpm and aeration was through sparger inlet. The amount of monomeric sugars remaining in the culture fluid was assessed by standard HPLC techniques. Culture supernatants were taken 24 h, and 48 h after inoculation.

Conditions used for carbon source exhaustion: Cultures were grown in batch fermentations in a BioFlo 3000 (New Brunswick Scientific) bioreactor with a 5-L working volume. Cultivations were performed with varying carbon source (glucose or xylose), nitrogen source (ammonium chloride or sodium nitrate), nitrogen concentration (low (282.4 mM) or high (564.8 mM)), and pH (4 or 5) (see Table 5). The medium composition, cultivation conditions and operating procedure of the bioreactor have been described in detail previously [35]. Samples for analysis of the carbon source concentration were collected every six hours and analyzed as described previously [35]. From each growth condition culture supernatants were taken after carbon source exhaustion.

**Table 5:** Overview of initial growth conditions used for carbon source exhaustion and time point of sampling

Experiment name	Carbon source	pH	Nitrogen source	Nitrogen source level (mM)	Sampling time (h)
*Nitrate, pH4*					
4 G 4NO_3_	Glucose	4	NaNO_3_	282.4	96
4 X 4NO_3_	Xylose	4	NaNO_3_	282.4	95
*Ammonium, pH4*					
4 G 8NH_4_	Glucose	4	NH_4_Cl	564.8	91
4 X 4NH_4_	Xylose	4	NH_4_Cl	282.4	85
*Nitrate, pH5*					
5 G 8NO_3_	Glucose	5	NaNO_3_	564.8	96
5 X 8NO_3_	Xylose	5	NaNO_3_	564.8	156
*Ammonium, pH5*					
5 G 8NH_4_	Glucose	5	NH_4_Cl	564.8	84
5 X 4NH_4_	Xylose	5	NH_4_Cl	282.4	90
5 X 8NH_4_	Xylose	5	NH_4_Cl	564.8	96

Analysis of total protein: The concentration of protein in cleared culture supernatants (or secretome enriched fractions) was measured by the Bio-Rad Protein Assay, using BSA as a standard. The procedure was fully automated using a COBAS MIRA Plus autoanalyzer.

### Liquid chromatography tandem mass spectrometric analysis

For secretome enriched fractions obtained from growth on sorbitol and galacturonic acid equal amounts of protein sample (250 μg) were separated on 12% SDS polyacrylamide gels, and stained with Colloidal Blue Staining (Invitrogen, Carlsbad, CA, USA). Gel lanes were cut into five slices, and each slice was treated with 50 mM dithiothreitol (DTT) in 50 mM NH_4_HCO_3 _(pH 8.0) for 1 h at 60°C. Next, slices were alkylated with 100 mM iodoacetamide in NH_4_HCO_3 _(pH 8.0) for 1 h at room temperature, washed with NH_4_HCO_3 _(pH 8.0). Slices were rehydrated in 10 ng/μl trypsin (Sequencing grade modified trypsin, Promega, Madison, WI, USA) and digested overnight at 37°C. LC-MS/MS conditions: samples were loaded on a preconcentration column and peptides were eluted to an analytical column with an acetonitrile gradient and a fixed concentration of formic acid. The resulting eluent was subjected to an electrospray potential *via *a coupled platinum electrode. MS spectra were measured on an LTQ-Orbitrap (Thermo Electron, San Jose, CA, USA) and MS scans of four most abundant peaks were recorded in data-dependent mode. To simplify the comparison between the two growth conditions the two galacturonic acid and the two sorbitol samples were pooled. Secretome enriched samples obtained from carbon source exhaustion were analysed with LC-ESI-MS-MS performed by Eurogentec (Seraing, Belgium). From each sample a volume corresponding to 10-15 μg of total protein was digested with trypsin, without prior separation of the proteins. To simplify the comparison with growth, all samples were pooled.

## Authors' contributions

MB and EM planned and performed the study. PJS performed the bioinformatics analysis. MB, EM and PJS wrote the paper with contributions from all authors. All authors read and approved the final manuscript.

## Supplementary Material

Additional file 1 ***Aspergillus niger *centred majority rule based classifier for signal peptide prediction validation**. *A. niger *centred orthologous protein clusters included in the classifier were selected for the presence of at least one putative SP protein. Click here for file

Additional file 2 ***Aspergillus niger *CBS 513.88 protein model re-annotation**. Re-annotation of five selected proteins with an ambiguous signal peptide prediction by alignment of the inferred proteins with orthologous *Aspergillus *proteins using the Muscle multiple sequence alignment tool. Click here for file

Additional file 3 *Aspergillus niger *proteins detected by high-throughput mass spectrometry of secretome enriched fractions cultured under a set of controlled conditions Click here for file
