# Supplementary material for: An inventory of the Aspergillus niger secretome by combining in silico predictions with shotgun proteomics data
Source: BMC Genomics. 2010 Oct 19;11:584. doi: 10.1186/1471-2164-11-584 (PMC3091731; doi:10.1186/1471-2164-11-584)
Supplement: Additional file 2 — Aspergillus niger CBS 513.88 protein model re-annotation. Re-annotation of five selected proteins with an ambiguous signal peptide prediction by alignment of the inferred proteins with orthologous Aspergillus proteins using the Muscle multiple sequence alignment tool. [file 1471-2164-11-584-S2.DOC]

**Additional file 2**. *Aspergillus niger* CBS513.88 protein model verification of four selected proteins with a false negative Signal Peptide predictions (example 1-4) and one protein with a false positive Signal Peptide prediction (example 5) by alignment of the inferred proteins with orthologous *Aspergillus* proteins using the Muscle **multiple sequence alignment tool.**

Example 1.

**An02g13580:** The GH18 (glycosyl hydrolase, family 18) type II chitinases hydrolyze chitin, an abundant polymer of beta-1,4-linked N-acetylglucosamine (GlcNAc) which is a major component of the cell wall of fungi (Signal peptide in red).

An02g13580 ----------Ms-LQCVASMRFAM----YHTsGLPGanQTQGITHAIMAFAQSTlFNSDS

ATCC_37548 --MKlIALSLMs-LQCVASMRFAM----YHTsGLPGanQTQGITHAIMAFAQSTlFNSDS

AO090020000207 MkwKsLALgLLAtaQsaASLRFvMYIDEYHTqGLPdSsgTaGIsHAvMgFAKSTlFNSDS

AFUA_3G07160 -mLKsLvfSLMA-vQaamgsRFAMYIDqYHTvdLPGSDQTQGvTHAIMAFApSkqFNSDS

AN11233 MiLKhaALALaA-LQCVAgLRFAMYIDEwHvnGLPGSDQTQGITHAIMgFAKSTdFtgDa

An02g13580 PPQFTPFEsVSTMRsRFSPgTKLMIAIGGWGDTSGFStaAKDEtSRnQYAKNVAAMVNQv

ATCC_37548 PPQFTPFEsVSTMRsRFSPgTKLMIAIGGWGDTSGFStaAKDEtSRnQYAKNVAAMVNQv

AO090020000207 PqswkPFEPidTMRKRFSsDTKLlvAIGGWGDTSGFSEGAKDEASRarYAKNVkAMVdEh

AFUA_3G07160 --sFTPFdtVnnMRKRFaPDTKvMIAIGGWGDTaGFSEGAKDEASRtkYAKNVAtMINnl

AN11233 PaaFqPFEPVSTfRnRFSPDTKvMIAIGGWGDsaGFSaGAKDEASRerYAKNVAAMlest

Example 2.

**An08g00490:** Putative gluconolactonase (Signal peptide in red).

An08g00490 -------------------------------------------------------MDtVT

ATCC_177169 MqrlLlLLasfTgsLvHAQTGVThPISEkCGRSVVCVNRYANVLPYHFFRNvS-TMDtVT

AO090005001626 -mawLqiLglvlsgLtHAQTGVTSPIAEaCGpSmVCiNRYANVLPYHFFRNvS-TMDdIS

AFUA_1G08990 MatailLLffaT---------VlSscgswdkRtViCVhkYAsVLPgkFsRtppinlgatg

An08g00490 TFGDTTVANGTvLQDVkTADFIVYnKEKGLDILGSNPSYEYvFAVNDAVHEAPVYVASQN

ATCC_177169 TFGDTTVANGTvLQDVkTADFIVYnKEKGLDILGSNPSYEYvFAVNDAVHEAPVYVASQN

AO090005001626 iFGDTTVASGTlLEgVnsANFlVYDRErGLEILGaNPSYkFMFAVseAVHEAPVYiASQN

AFUA_1G08990 SFasavVpadTsfasianAtFIVYDlaraqaIrGSaPtfQmMlA------------rpws

Example 3.

**An11g03120:** Putative xylosidase/arabinosidase (Signal peptide in red).

An11g03120 --------------------------MsHPq---QNlLaTttSN---------tKAGNPV

ATCC_179682 mkhhnyyPstclsILpfLL----plTMsHPq---QNlLaTttSN---------tKAGNPV

AO090003000239 ----MhlkPIqtllpTLLL----flhLptPalttQsPLsps--t---------PKsGNPV

AFUA_2G04480 ----MktPPIpihILTLtListtltSaatPtl--QsPLeSpdnNalnnslnmnPKtGNPi

AN1870 ----------mgrILSLLL----laSfvsale--QdPLvSeats---------alAGNPV

An11g03120 FPGWYADPEARlFNAQYWIYPTYSAdYsEQTFFDAFSSPDLLTWTKHPTILNiTnIPWST

ATCC_179682 FPGWYADPEARlFNAQYWIYPTYSAdYsEQTFFDAFSSPDLLTWTKHPTILNiTnIPWST

AO090003000239 inGWYADPEARIFdttYWlYPTYSAAYEaQTFFDAFSSPDLLTWTKHPTILNlTaIPWST

AFUA_2G04480 iqGWYADPEARIFdttYWIYPTYSAAYEsQTFFDAFSSqDLrTWTKHPrILeFgGIPWST

AN1870 FPGWYADPEARIFNdQYWIYPTYSAAYEEQTFFDAFSSPDLLTWTKHPaILNFsGIPWST

Example 4.

**An15g05750:** Putative Golgi protein of unknown function (DUF1753). The predicted gene-models result in an over extended N*-*terminal region (Signal peptide in red).

An15g05750 MGqriggdhrgidrradrpenrapvkpaigrQrhLnTpgcvlFfqsRspaspSipFplsv

ATCC_53494 MG--------------------------l------------------------pRl----

AO090012000272 MG--------------------------ivtQllLtTsllpfFsseRigfhfrfpF----

AFUA_2G15580 MG--------------------------F-----------------------SaRF----

AN7601.2 MG--------------------------F-----------------------SsRF----

An15g05750 apflppptppthpiLnpPpLhniPsavisllgdinhgfipspphpaargwepdpvwtpva

ATCC_53494 --------------LRiPR----P------------------------------------

AO090012000272 --------------LlhPpIacat------------------------------------

AFUA_2G15580 --------------LRvPR----P------------------------------------

AN7601.2 --------------LRiPR----P------------------------------------

An15g05750 rsraemrltiasrlsgdltQTFLYVMSLQTGASLITLSLLLNKISGLYGLLALLTGYHLS

ATCC_53494 -------------------ETFLYVMSLQTGASLITLSLLLNKISGLYGLLALLTGYHLS

AO090012000272 -------------------pTaahrryqrwGshqpvfSasrgq-SGLYGLLALLTGYHLS

AFUA_2G15580 -------------------ETFLYVMSLeTGASLITLSLLLNKISGLYGLLALLTGYHLS

AN7601.2 -------------------QTFLYVMSLQTGASLITLSLLLNKISGLYGLLALLTGYHLS

An15g05750 PVQLSMYiYSLLALGLATLLFPHIRKQtPLQCLALAWLYVFDSLINAAYTAAFGVTWFLV

ATCC_53494 PVQLSMYiYSLLALGLATLLFPHIRKQtPLQCLALAWLYVFDSLINAAYTAAFGVTWFLV

AO090012000272 PVQLSMYLYSLLALGVATLLFPHIRKQSPLQCLALAWLYlFDSVINAAYTAAFGVTWFLV

AFUA_2G15580 PVQLSMYLYSLiALGLtayLFPHIRKQSPLQCLALAWLYllDtVINAAYTAAFGVTWFLV

AN7601.2 PVQLSMYLYSLiALastvLLFPHIRKQSPLQCLALAWLYVFDSaINAAYTAAFGVTWFLV

Example 5.

**An15g01200:** FAD/FMN-containing isoamyl alcohol oxidase. The protein sequence of both An15g01200 and the ATCC equivalent 137591 should be N-terminal extended. A screen of ATCC1015 FLX EST sequence data (http://www.broadinstitute.org/)) indeed reveals an alternative start codon upstream of the assigned start codon with a signal anchor probability of 0.993 (CBS false positive Signal Peptide prediction in red). CBS/ATCC_new presents the alternative start codon of genemodel An15g01200/ATCC_137591 based upon the EST screen.

CBS/ATCC_new MFNPISKLVRRGDSSGSNGDLSPTMVDLLISLLVLILLGlALVgALLVLRRKRLNREQSEL

An15g01200 -----------------------MVDLLISLLVLILLGlALVgALLVLRRKRLNREQSEL

ATCC_137591 ------------DSgsNDGLSPtMVDLLISLLVLILLGlALVgALLVLRRKRLNREQSEL

AFUA_6g03620 mfiKLVtRgdSsnSddNnsLaPAMiDLLISLLVLIiLGIvLVatLLVLRRKRqNqmdSEL

AN6588 -mnQIMhQffArDdakdDGLSeSMVDLLISLLVLvLLGIvLictLLVLRRrRLNRQQSEL

An15g01200 PVHNGQCvPNHRRFTvSASPNAKTESVLVYDEKRSLIENSSSPPPSPVPEIRITFPEEED

ATCC_137591 PVHNGQCvPNHRRFTvSASPNAKTESVLVYDEKRSLIENSSSPPPSPVPEIRITFPEEED

AFUA_6g03620 PVHNGQCsPNHRRlTISASsNsrTqSiLVYDEKRSLIENSSSPPPSPVPEIRITFPEEED

AN6588 PVHNGQCsthHRsvTISApPyAKTESVfViDEKRnLmENSSSPPPSPVPEIRITFPEEED
